# Complete mitochondrial genome of *Penthimia melanocephala* Motschulsky, 1863 (Hemiptera: Cicadellidae: Deltocephalinae)

**DOI:** 10.1080/23802359.2021.1875905

**Published:** 2021-02-12

**Authors:** Tie-Long Xu, Ren-Huai Dai

**Affiliations:** Institute of Entomology, Guizhou University, The Provincial Key Laboratory for Agricultural Pest Management Mountainous Region, Guiyang, Guizhou, PR China

**Keywords:** Mitogenome, *Penthimia melanocephala*, phylogeny

## Abstract

*Penthimia* is the largest genus in the tribe Penthimiini of the subfamily Deltocephalinae. To date, there are no available mitogenome sequences from *Penthimia*. In this study, we have sequenced and annotated the complete mitogenome of *Penthimia melanocephala* (Motschulsky 1863), the mitogenome is 15,308 bp in length, which including 13 protein-coding genes (PCGs), 2 ribosomal RNA genes (rRNAs), 22 transfer RNAs (tRNAs) and a long non-coding region (control Region), base composition of whole sequences are A (50.4%), C (15.3%), G (10.0%), and T (24.3%). All PCGs started with the typical ATN codon and stopped with the typical TAN codon except for *COII* and *COIII* stopped with single T. Within phylogenetic tree, Deltocephalinae members were clustered into a clade. The complete mitogenome of *P. melanocephala* can provide essential DNA molecular data for further evolutionary and phylogenetic analysis.

The genus of *Penthimia* was established by Germar in 1821, it is the largest genus in the tribe Penthimiini of the subfamily Deltocephalinae. To date, there are no available mitogenome sequences from *Penthimia*. *Penthimia melanocephala* was established by Motschulsky in 1863 and it was reviewed based on the examination of type specimens by Shobharani et al. (2018). To better understand the diversity and phylogeny of Cicadellidae, the complete mitochondrial genome of *P. melanocephala* was first reported in this article.

*Penthimia melanocephala* from China was recorded and described by Li and Wang (1991). Our samples were collected by sweep net from Mt. Huanglian, Yunnan Province of China (102°18′32″ E, 22°53′49″ N), in June 2019. The species identification was conducted by looking up the relevant literature (Shobharani et al. 2018). Male external genitalia and genome DNA are deposited in the Institute of Entomology, Guizhou University, Guizhou Province, China (GUGC-HCD-00114).

The complete mitogenome sequences of *P．melanocephala* were determined using next-generation sequencing method for the first time. All *tRNA* genes are identified by ARWEN version 1.2 software (Laslett and Canbäck 2008). The phylogenetic relationships of *P. melanocephala* were reconstructed by the maximum likelihood method with MEGA version 7 software, 1000 replicates of bootstrap were set. Each protein-coding gene (PCG) sequences were aligned using the MAFFT algorithm in TranslatorX and aligned sequences were eliminated using Gblocks 9.1b (Abascal et al. 2010).

The complete mitogenome of *P. melanocephala* is 15,038 bp in length (GenBank Accession Number: MT768010), which including 13 PCGs, 2 ribosomal RNA genes (rRNAs), 22 transfer RNA genes (tRNAs), and 1 major noncoding region referred to as the control region, base composition of whole sequences are A = 50.4%, C = 15.3%, G = 10.0%, T = 24.3%, the A + T content of the whole mitogenome was 74.7%, which is within the range reported from Hemipteran mitogenomes (68.86–86.33; Wang et al. 2015). Total length of 13 PCGs is 10,943 bp, encoding 3636 amino acids. As the longest gene, the *ND5*, which has containing 1680 bp, locates between *trnF* and *trnH*. Between *COIII* and *ND3*, there is the shortest gene which is *trnG*, the length of trnG is 60 bp. Furthermore, the position of the control region is between *srRNA* (12S) and *trnI* and the control region contained 1007 bp. The *lrRNA* (16S) located between *trnL2* (CUN) and *trnV* contains 1180 bp, the *srRNA* (12S) containing 737 bp located between *trnV* and control region. All PCGs started with the typical ATN codon and stopped with the typical TAN codon except for *COII* and *COIII* stopped with single T.

Phylogenetic analysis using nucleotide sequences of 13 PCGs was conducted with 22 species of Membracoidea from 11 subfamilies. The available mitogenome sequences of 21 species downloaded from GenBank and two species of Membracidae as outgroups. Result clearly indicated that *P. melanocephala* and *Reticuluma hamata* were clustered into a clade and *P. melanocephala* belong to Penthimiini of Deltocephalinae (Figure 1). Currently, only one study has been recorded for Penthimiini (Xu and Dai 2020), and this study provides molecular data for further phylogenetic and evolutionary analysis for Deltocephalinae.

**Figure 1.**
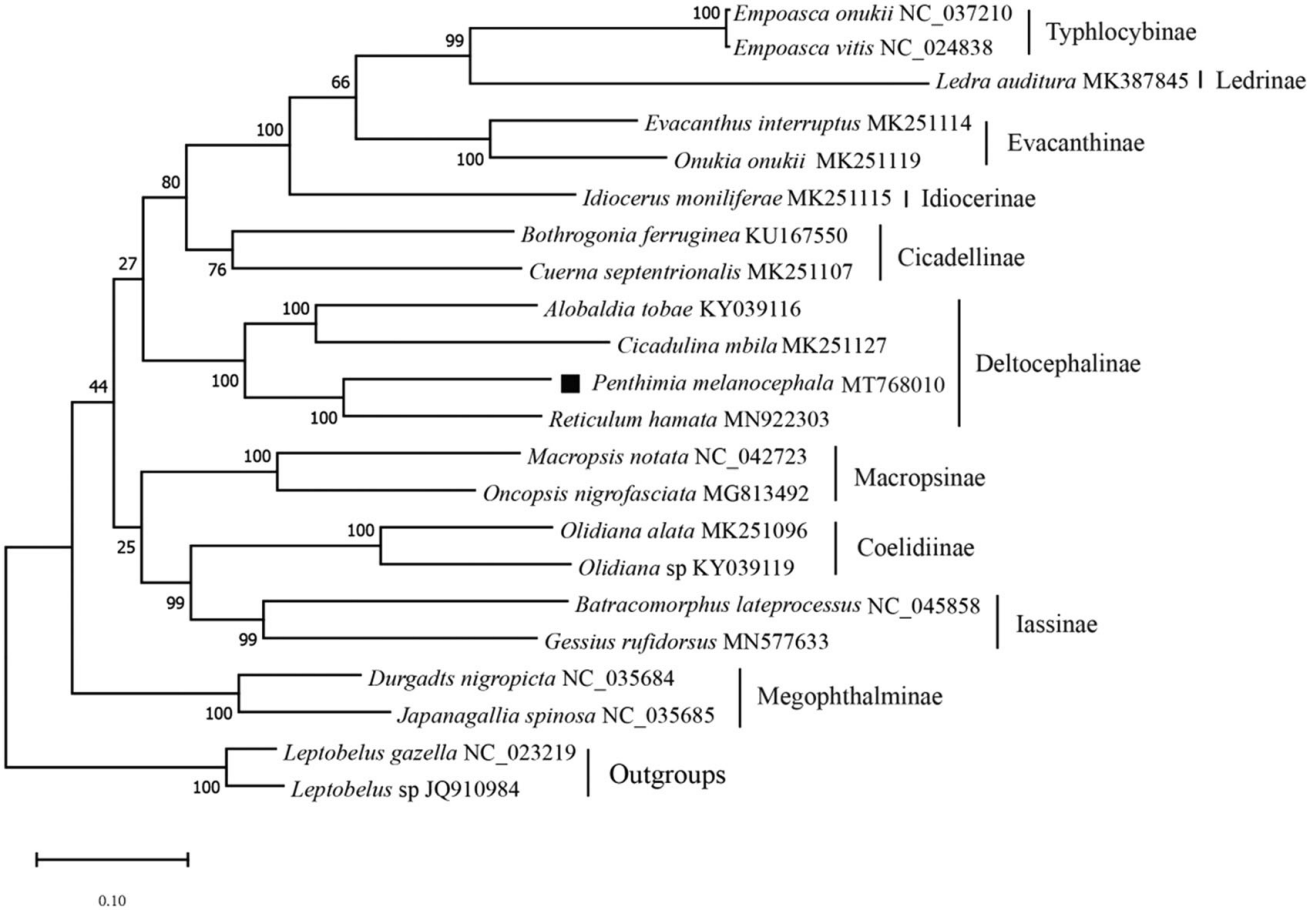
Maximum-likelihood phylogeny based upon the concatenated the nucleotides of the 13 PCGs of 19 ingroup species and 2 outgroup species by MEGA7. The accession number for each species is indicated after the scientific name.

## Data Availability

The data that support the finding of this study are openly available in NCBI at (https://www.ncbi.nlm.nih.gov), reference number [MT768010].
